# Emerging technologies and current challenges in intratumoral microbiota research

**DOI:** 10.3389/fcimb.2025.1685862

**Published:** 2026-01-02

**Authors:** Zhiyue Wang, Tianqi Zhang, Yang Liu

**Affiliations:** 1 Department of Hepatobiliary and Pancreatic Surgery Division, Ningbo No.2 Hospital, Ningbo, China; 2 Ningbo Key Laboratory of Intestinal Microecology and Human Major Diseases, Ningbo, China

**Keywords:** anaerobic bacteria, artificial intelligence, intratumoral microbiota, organoid models, tumor microenvironment

## Abstract

Intratumoral microbiota are now recognized as an integral component of the tumor microenvironment, affecting tumor initiation, metastatic potential, immune modulation, and treatment response. However, their extremely low biomass poses significant challenges for accurate detection, functional interpretation, and reproducibility, largely because the detection process is highly susceptible to environmental contamination. Standardization of analytical procedures has not yet been established; consequently, variability in sampling protocols, sequencing workflows, and bioinformatic pipelines further complicates cross-study comparisons and hampers the consolidation of robust evidence in this field.

Recent advances in technology have begun to provide opportunities to overcome these barriers. Improved contamination-control strategies and more sophisticated decontamination algorithms have enhanced the reliability of microbial detection in low-biomass tissues. High-resolution approaches, such as single-cell RNA sequencing, spatial transcriptomics and optimized anaerobic cultivation, enable the sensitive identification, spatial localization, and mechanistic study of tumor associated microbes. Parallel developments in genome-resolved and enzyme-level analysis reveal microbial metabolic pathways that shape immune responses, drug resistance, and tumor progression. Organoid-based co-culture models further provide physiologically relevant platforms to dissect host-microbe-immune interactions and interpret microbiota-driven modulation of therapeutic responses. Integrating microbiome data with clinical and multi-omics profiles, assisted by artificial intelligence, is accelerating biomarker discovery and informing microbe-guided therapeutic strategies.

Taken together, the standardization of research strategies, combined with the application of advanced detection technologies, is propelling the field beyond descriptive profiling toward mechanistic understanding and clinical translation, thereby unlocking the potential of intratumoral microbiota for precision oncology.

## 1 Introduction

The historical assumption that solid tumors represent a sterile compartment has been decisively overturned. Independent investigations employing orthogonal methodologies have now demonstrated that most human malignancies harbor resident microbial communities comprising bacteria, fungi, and viruses, many of which reside intracellularly within neoplastic and immune cells (Nejman et al., 2020; Fu et al., 2022). These intratumoral microbiota are increasingly recognized as active modulators of tumor initiation, progression, immune surveillance, metastatic dissemination, and therapeutic response (Hou et al., 2025; Quan et al., 2025).

In contrast to the high microbial biomass characteristic of the gastrointestinal tract or cutaneous surfaces, intratumoral populations typically exist at abundances several orders of magnitude lower, frequently approaching or falling below the detection threshold of conventional sequencing platforms (Tiğli and Gümüşderelioğlu, 2008). This extreme paucity of microbial nucleic acids renders studies exquisitely susceptible to contamination and technical noise, while pronounced spatial heterogeneity within individual tumors and compositional variation across cancer types further confound interpretation. Consequently, the majority of published work remains largely descriptive, focused on taxonomic enumeration rather than functional characterization. Systematic interrogation of microbial metabolism, host–microbe cellular crosstalk, and the behavior of the predominant obligate anaerobes remains limited (Weyrich et al., 2019; Stocker et al., 2020; Fu et al., 2023).

Particular attention has been directed toward immune–microbiota interactions within the tumor microenvironment. Accumulating evidence indicates that intratumoral bacteria can reprogram myeloid and lymphoid compartments, alter checkpoint-inhibitor efficacy, and influence clinical outcome (Xie et al., 2022; Lu et al., 2024; Doyle et al., 2025; Ke et al., 2025). These organisms engage in complex, bidirectional interactions with cancer cells, fibroblasts, and immune effectors, establishing regulatory networks that shape local and systemic anti-tumor immunity (Gao et al., 2025; Xue et al., 2025; Zhang et al., 2025). Dissecting taxon-specific contributions within such multifaceted ecosystems, however, demands experimental systems capable of recapitulating physiological microbial loads and three-dimensional tissue architecture—requirements increasingly met by immune-competent patient-derived tumor organoids (Martins et al., 2021).

Rapid technological progress in spatial transcriptomics, single-cell microbiomics, anaerobic cultivation platforms, and computational decontamination now provides the resolution and specificity required to move beyond correlation toward causal inference (Gao et al., 2025). When coupled with rigorous standardization and interdisciplinary collaboration, these advances position intratumoral microbiota research to redefine fundamental paradigms in tumor biology and to inform novel diagnostic and therapeutic strategies in clinical oncology.

## 2 Challenges in intratumoral microbiota research: low biomass, contamination risks, and lack of standardization

Investigation of microbial communities residing within solid tumors differs fundamentally from the study of high-biomass ecosystems such as the intestinal or cutaneous microbiota. The microbial burden in most tumors is extraordinarily low, typically ranging from 10 to 10^4^ bacterial genomes per million human cells, a level that approaches or falls below the reliable detection limit of standard molecular workflows (Nejman et al., 2020; Fu et al., 2022; Lu et al., 2024). This paucity of endogenous microbial nucleic acids creates pervasive technical obstacles that affect every phase of analysis, from initial detection and quantification to downstream functional and translational studies.

Current methodologies frequently lack the sensitivity and specificity required for confident profiling of such communities. Bulk sequencing of tumor homogenates yields datasets dominated by stochastic sampling effects, host-derived sequences, and extraneous contaminants, resulting in incomplete taxonomic inventories and potentially spurious associations between microbial features and clinicopathological variables (Choi et al., 2019). Compounding this issue, microbial colonization within a single tumor exhibits marked spatial heterogeneity, with organisms often restricted to hypoxic necrotic cores, perivascular niches, or intracellular compartments (Ke et al., 2025). Compositional differences across tumor types, between patients with identical histology, and even between primary and metastatic lesions in the same individual further erode reproducibility and generalizability.

The low endogenous signal also severely constrains mechanistic interrogation. Establishing causality, whether a given taxon actively drives phenotypic changes or merely persists as a bystander, requires experimental systems in which microbial abundance, viability, and host response can be precisely controlled (Baldrian and López-Mondéjar, 2014; Zhou et al., 2024). In clinical specimens, however, microbial DNA is frequently at or below the level of background contamination, rendering conventional infection models and functional validation approaches impractical.

From a translational perspective, these limitations currently preclude the development of robust intratumoral microbiota-based biomarkers. Profiles that cannot be reproducibly measured within or between laboratories are unsuitable for diagnostic, prognostic, or predictive applications. Until substantial improvements in sensitivity, spatial resolution, and standardization are achieved, clinical implementation will remain unfeasible.

### 2.1 Low microbial biomass in intratumoral samples increases the risk of contamination

The scarcity of genuine microbial nucleic acids renders tumor samples uniquely susceptible to distortion by extraneous DNA introduced at any stage of the workflow, from surgical resection to library preparation and sequencing (Schorr et al., 2023). Even trace contamination originating from skin flora, operating-theatre air, laboratory surfaces, or molecular-biology-grade reagents can constitute the dominant signal in the final dataset, generating false-positive taxa or masking authentic residents (Cao et al., 2024). Multiple high-profile studies have required correction or retraction after rigorous negative controls revealed that reported microbial signatures were artefact-derived.

Effective mitigation demands comprehensive contamination-control strategies: certified nuclease-free consumables, dedicated pre-PCR laboratory space, multiple blank controls processed in parallel with every experimental batch, and deep sequencing of those blanks to establish background profiles. Despite the adoption of such measures by leading groups, substantial inter-laboratory variation in protocols persists, undermining cross-study comparability and sustaining legitimate skepticism regarding the biological significance of many published findings (Xie et al., 2022).

### 2.2 Major contamination issues in low microbial biomass studies

In low-microbial-biomass contexts, even minute quantities of extraneous DNA can profoundly distort the observed community structure. The predominant sources, repeatedly identified through systematic negative-control sequencing, are as follows:

a. Environmental contamination: During tumor resection, specimen transport, and pathological grossing, tissue is exposed to operating-theatre air, gloved hands, and cutting surfaces. Common skin commensals (e.g., *Cutibacterium acnes*, *Staphylococcus epidermidis*, *Streptococcus* spp.) and airborne environmental bacteria (e.g., *Micrococcus* spp., *Bacillus* sp*ores*) are routinely detected at high relative abundance when appropriate blanks are omitted (Ge et al., 2025). In high-biomass specimens, these inputs are biologically negligible; in tumor samples, they frequently constitute the majority of recovered sequences.
b. Reagent contamination: Commercial DNA-extraction kits, polymerases, and master mixes are manufactured under non-sterile conditions and consistently harbor trace bacterial DNA, predominantly from water- and soil-associated genera (*Pseudomonas*, *Ralstonia*, *Bradyrhizobium*, *Acinetobacter*, *Stenotrophomonas*). Independent surveys have documented highly reproducible “kit-ome” signatures that persist across manufacturers and production lots (Doyle et al., 2025). In tumor specimens, where endogenous microbial DNA may be orders of magnitude lower, this background signal is readily misinterpreted as tumor-specific microbiota.
c. Across samples contamination: On Illumina patterned-flow-cell instruments, imperfect demultiplexing can cause 0.1–2% of reads to be assigned to the incorrect barcode. Although inconsequential in high-biomass libraries, this leakage is sufficient to transfer hundreds or thousands of reads from a high-biomass positive control or unrelated sample into an ultra-low-biomass tumor library, fabricating entire apparent communities (van der Valk et al., 2020).
d. Signal noise: Many computational decontamination algorithms rely on prevalence or inverse abundance-concentration relationships to identify contaminants. When authentic intratumoral taxa are themselves rare and heterogeneously distributed, their statistical behavior closely resembles that of background contaminants. Overly stringent filtering risks removal of genuine biological signal, whereas lenient thresholds retain laboratory-derived artefacts (Tiğli and Gümüşderelioğlu, 2008). This inherent overlap remains one of the most intractable challenges in the field.

### 2.3 Lack of standardization and reproducibility issues in low microbial biomass studies

Reproducibility represents one of the most serious unresolved issues in low-microbial-biomass microbiome research, and intratumoral studies are particularly affected. When identical tumor specimens are divided and processed independently (either in different laboratories or in separate batches within the same facility), the resulting microbial profiles frequently exhibit profound discrepancies (Eisenhofer et al., 2019; Rajar et al., 2022). The principal sources of this technical variability include:

#### 2.3.1 Differences in DNA extraction protocols

Mechanical disruption method, lysis buffer composition, enzymatic cocktails, and purification chemistry (silica columns versus magnetic beads) all exert taxon-specific effects on recovery efficiency, especially for Gram-positive, spore-forming, or anaerobic organisms that predominate in many tumors (Anwar et al., 2025; Rauer et al., 2025).

#### 2.3.2 Primer selection and PCR conditions

Commonly employed 16S rRNA hypervariable regions (V1–V3, V3–V4, V4, V4–V5) display well-documented phylogenetic biases. Minor variations in annealing temperature, cycle number, polymerase fidelity, or chimera removal stringency can dramatically alter apparent community composition.

#### 2.3.3 Sequencing platforms and depth

Illumina, PacBio, and Oxford Nanopore platforms differ in error profiles, read length, and GC bias. Inadequate sequencing depth exacerbates stochastic undersampling of rare taxa, whereas platform-specific artefacts introduce systematic distortion.

#### 2.3.4 Bioinformatics pipelines and reference databases

Denoising algorithm (DADA2, Deblur, UNOISE3), clustering threshold, taxonomic classifier (RDP, SILVA, Greengenes2, GTDB), and contamination-removal strategy routinely yield non-concordant results from identical sequence data (Bharti and Grimm, 2021). Incomplete or inconsistently curated reference databases further compromise species- and strain-level assignment of tumor-associated anaerobes.

#### 2.3.5 Batch effects compounded by variable contamination

Because background contaminant communities themselves differ between reagent lots, laboratory environments, and sequencing runs, technical and biological sources of variation become inextricably confounded (Rodriguez et al., 2025).

Large-scale inter-laboratory ring trials and re-analyses of shared datasets have repeatedly demonstrated that altering any single parameter can shift a taxon from statistically significant enrichment to undetectable, or vice vers (Rodriguez et al., 2024). At present, no internationally endorsed standard operating procedures exist for specimen collection, storage, nucleic-acid extraction, library preparation, sequencing, or data analysis in the intratumoral microbiome field. The absence of such harmonized, ring-tested protocols severely restricts meta-analysis, hinders biomarker discovery, and sustains legitimate skepticism regarding the robustness of many published microbial signatures.

Until the community establishes and enforces consensus guidelines—ideally through coordinated efforts analogous to the Human Microbiome Project or the International Cancer Microbiome Consortium—reproducibility will remain a critical barrier to scientific progress and clinical translation.

### 2.4 Landmark discoveries and remaining limitations in intratumoral microbiome research

Despite the formidable technical obstacles outlined above, a series of landmark studies has unequivocally established the existence and potential biological significance of intratumoral microbiota. Pushalkar et al. provided early definitive evidence in pancreatic ductal adenocarcinoma, demonstrating that tumor-specific bacterial and fungal communities accelerate oncogenesis through toll-like-receptor-mediated immune suppression in genetically engineered mouse models (Pushalkar et al., 2018). Nejman et al. subsequently conducted the largest cross-cancer survey to date, analyzing over 1,500 tumors from seven malignancies and revealing tumor-type-specific intracellular bacterial signatures in both the neoplastic and immune compartments (Nejman et al., 2020). More recent investigations have progressed from description to function: intracellular *Fusobacterium* and other anaerobes have been shown to enhance metastatic fitness in breast cancer by cytoskeletal remodeling that protects circulating tumor cells from fluid shear stress (Fu et al., 2022), while spatially restricted bacterial consortia in colorectal and oral carcinomas correlate with immunosuppressive microenvironments and attenuated response to immune-checkpoint blockade.

These studies have collectively shifted consensus from questioning the very existence of intratumoral microbes to accepting them as structured, reproducible components of the tumor ecosystem. Nevertheless, they were conducted against a backdrop of persistent methodological constraints that continue to limit interpretive strength and generalizability.

Sample sizes remain modest relative to tumor heterogeneity, biopsies capture only a minute fraction of total tumor volume, and most profiling has relied on 16S rRNA amplicon sequencing or shallow shotgun metagenomics—approaches that offer limited strain-level resolution and no direct insight into viability or metabolic activity. Contamination-control standards, although markedly improved in the most recent publications, were inconsistent in earlier work, and many datasets lack the comprehensive blank sequencing now regarded as obligatory. Consequently, although the presence of intratumoral bacteria is no longer credibly disputed, the precise taxonomic composition, intracellular versus extracellular localization, viable fraction, and—most critically—the causal contribution of individual species or consortia to tumor behavior remain incompletely resolved.

## 3 Contamination control and data processing in low biomass tumor microbiome research

The validity of any intratumoral microbiome investigation ultimately rests on the ability to demonstrate, beyond a reasonable doubt, that detected microbial sequences originate from within the tumor rather than from extraneous sources. In the context of extremely low endogenous biomass, this demands an uncompromising, multi-tiered strategy that integrates meticulous wet-laboratory controls, comprehensive negative-control sequencing, and rigorous computational decontamination. The recommendations presented below reflect the current consensus of leading low-biomass and tumor-microbiome laboratories and are now widely regarded as the minimum acceptable standard for credible publication and clinical translation.

### 3.1 Recommendations for minimizing and identifying contamination in study design and controls

**Figure 1:**
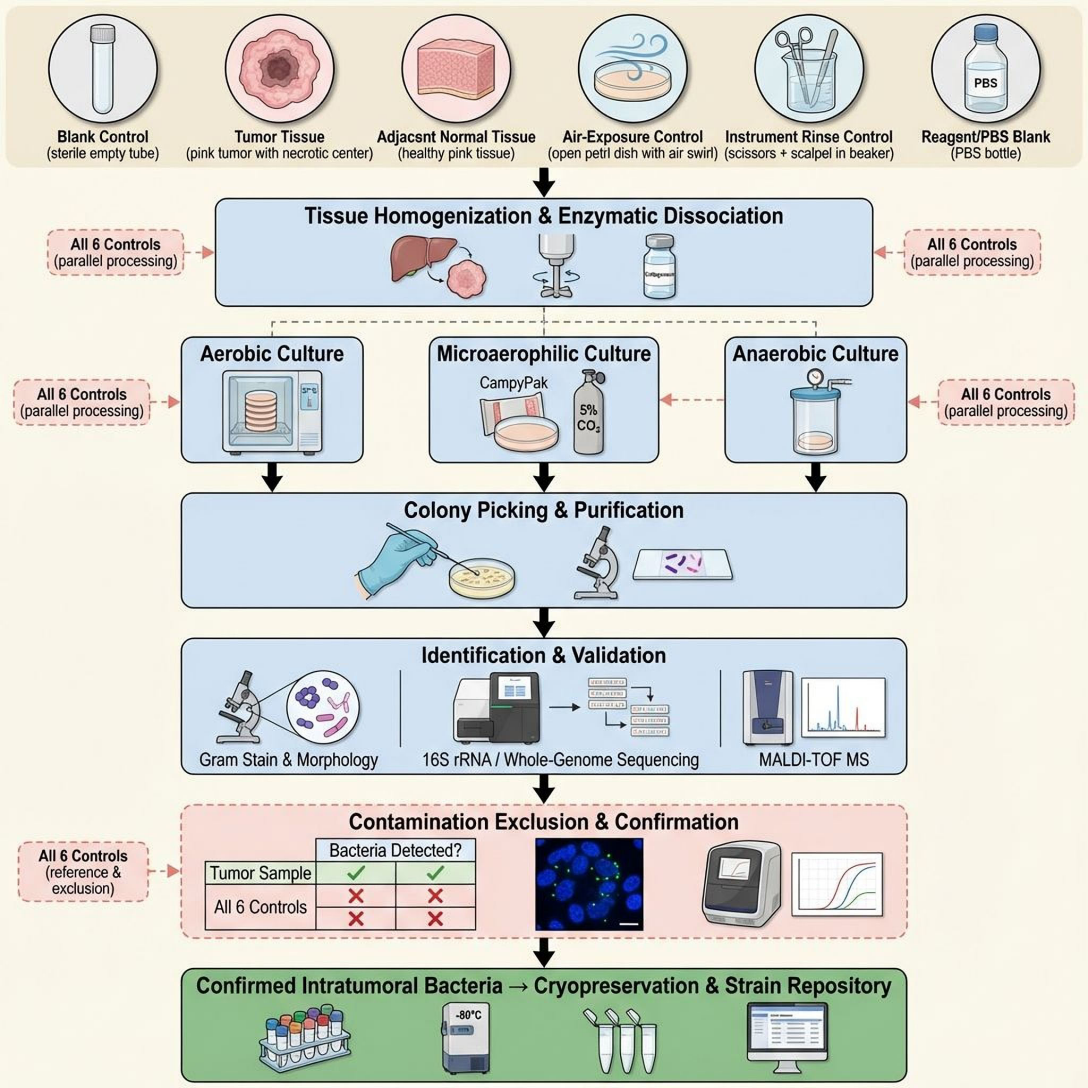
Schematic diagram of the complete workflow for isolation, culture, and contamination exclusion of intratumoral bacteria under strict aseptic conditions. Tumor tissue and five negative controls (blank control, adjacent normal tissue, air-exposure control, instrument rinse control, reagent/PBS blank) were collected simultaneously. Following tissue homogenization and enzymatic dissociation, samples underwent primary culture under microaerophilic conditions and anaerobic conditions. Positive colonies underwent repeated picking and purification, followed by Gram staining and morphological observation, 16S rRNA or whole-genome sequencing, MALDI-TOF mass spectrometry (MALDI-TOF MS), and qPCR to compare bacterial abundance between tumors and negative control groups (Tumor vs Controls). Only samples meeting strict contamination control standards are designated as Confirmed Intratumoral Bacteria, proceeding to cryopreservation and strain repository storage. Otherwise, they are classified as Contaminants and discarded. This workflow systematically excludes contamination from skin, environment, instruments, and reagents through multiple sampling controls, culture condition controls, molecular identification, and quantitative validation, ensuring the authenticity and reproducibility of intratumoral bacterial isolation results.

Environmental, negative, and positive controls represent best practices for working with low-biomass materials. Comparing these control groups helps to identify and remove contaminants and validate the associations of intratumoral microbes and tumor-type-specific microbial signatures (Fu et al., 2022). Notably, the composition of the human tumor microbiome varies according to tumor type (Fu et al., 2023). Moreover, to maintain experimental rigor, negative controls should be incorporated at each step of the workflow, including sample collection, nucleic acid extraction, and PCR. Implementation of stringent as well as comprehensive contamination control measures is essential because of the extremely low-biomass of intratumoral microorganisms (**Figure 1**).

a. Blank Controls (Negative Controls): Blank control groups do not have tissue but do get the same experimental procedures as other tissue samples and the same extraction process (Weyrich et al., 2019). Such controls are to flag contamination from reagents, labware, equipment, and the laboratory environment.
b. Environmental Controls: Samples collected from the laboratory (e.g., air, equipment surfaces) may be included with the samples or separately to detect contamination occurring during sampling or testing.
c. Instrument Controls: Samples from surgical instruments serve as controls to rule out contamination of the tumor microbiome introduced before tumor removal. This control group helps identify potential contamination from the operating room environment.
d. Positive Controls: Positive controls can include specific bacterial strains of interest and tissues that have been verified to host bacteria, such as colorectal tumor specimens (Iwaszkiewicz-Eggebrecht et al., 2024). These controls are essential to ensure the accuracy and sensitivity of methods for detecting and identifying bacteria.

### 3.2 Data processing methods to exclude contamination in low microbial biomass studies

a. Distinguishing true microbial signal from contaminants: Direct comparison of amplicon sequence variant (ASV) or operational taxonomic unit (OTU) tables between tumor specimens and matched negative controls remains the cornerstone of contaminant identification. Sequences that occur at comparable or higher relative abundance in extraction blanks, PCR negatives, or environmental controls are flagged and removed prior to downstream analysis (Xue et al., 2025).

b. Statistical identification and removal of contaminants: Sample contamination can be identified using tools such as Decontam and microDecon, which detect contaminant sequences or OTUs by analyzing their relative abundance in control samples and their correlation with DNA concentration (Zhang et al., 2025). Frequency-based filtering detects sequences inversely correlated with total DNA input, while prevalence-based filtering flags sequences abundant in negative controls but rare in actual samples (**Table 1**). Decontam employs two complementary algorithms: (i) a prevalence-based method that identifies taxa significantly enriched in negative controls, and (ii) a frequency-based method that detects sequences whose abundance inversely correlates with total input DNA concentration across samples. Both approaches are implemented within the widely used phyloseq framework and generate probabilistic contaminant classifications (Yajima et al., 2018).

**Table 1:** Overview of decontam and microdecon.

Feature	Decontam	MicroDecon
Primary role	Statistical contamination classification	Quantitative contamination subtraction
Input	ASV/OTU table + metadata	ASV/OTU table + negative controls
Output	Contaminant probabilities, labels	Decontaminated abundance matrix
Method	Statistical classification	Quantitative subtraction
Resolution	Contaminant identification	Contaminant correction
Strengths	Flexible, interpretable, and integrates with phyloseq	Quantitative correction, intuitive interpretation
Limitations	Relies on concentration data or many blanks	Highly dependent on blank representativeness
Best used for	Removing contaminants in microbiome data	Quantitative correction of contamination

MicroDecon applies a quantitative subtraction algorithm that proportionally removes the taxonomic profile observed in blank controls from each biological sample, thereby preserving low-abundance genuine taxa that would otherwise be eliminated by binary removal (Inamdar and Pulinthanathu, 2019).

c. Host DNA filtering: Due to the high abundance of host DNA in tumor samples, it is essential to rigorously remove host-derived sequences through computational methods. Tools such as Bowtie2 or Kraken2 (**Table 2**) can be used to align sequencing reads to the host genome, enabling the exclusion of host DNA and thereby enhancing the sensitivity and accuracy of microbial detection (Gao et al., 2025).

**Table 2:** Overview of Bowtie2 and Kraken2.

Feature	Bowtie2	Kraken2
Primary role	Read alignment	Taxonomic classification
Input	FASTQ reads, index files	FASTQ reads, reference database
Output	SAM/BAM alignments	Taxonomic classification report
Method	Burrows-Wheeler Transform (BWT)	Exact k-mer matching with LCA
Resolution	Nucleotide-level mapping	Taxonomic-level assignment
Strengths	High accuracy in mapping to the reference genome; efficient host read removal	Fast, efficient taxonomic profiling
Limitations	Slow with large datasets; sensitive to reference genome completeness	Less accurate at lower taxonomic levels (e.g., species)
Best used for	Mapping reads to the reference genome, host removal	Profiling microbial community composition at multiple taxonomic levels

Bowtie2 rapidly aligns reads against the human reference genome and excludes mapped sequences (Langmead and Salzberg, 2012).

Kraken2, using k-mer-based classification against a combined human-microbial database, offers greater speed and sensitivity when the microbial signal is extremely weak (Wood et al., 2019).

d. Batch effect and systematic contamination analysis: Principal coordinate analysis, hierarchical clustering, or supervised methods (e.g., PERMANOVA) are routinely applied to visualize and quantify batch effects. Established correction algorithms such as ComBat-seq or removeBatchEffect are then used to mitigate systematic technical variation without distorting the biological signal (Martins et al., 2021).

e. Integration into microbiome analysis pipelines: Incorporate contaminant detection, host DNA filtering, and batch correction steps into common microbiome workflows like QIIME2, DADA2, and Phyloseq (**Table 3**). Performing these steps early during quality control ensures cleaner datasets for downstream analyses (Panzer et al., 2023).

**Table 3:** Overview of QIIME2, DADA2, and Phyloseq.

Feature	QIIME2	DADA2	Phyloseq
Primary role	End-to-end microbiome analysis	ASV inference and denoising	Downstream statistical analysis and visualization
Input	Raw reads (FASTQ), metadata	Raw reads (FASTQ)	Feature table (ASV/OTU), taxonomy, metadata, phylogeny
Output	Feature table, taxonomy, diversity metrics, visualizations	ASV table, error model, chimera-free sequences	Diversity metrics, ordinations, plots
Method	Plugin-based workflows (can include DADA2, Deblur)	Probabilistic error modeling	Data integration and R-based analysis
Resolution	ASV (via DADA2/Deblur) or OTU	ASV (single-nucleotide)	Depends on input (ASV or OTU)
Strengths	End-to-end, reproducible, plugin ecosystem	High-resolution, error-corrected ASVs	Flexible R analysis, rich visualization, integration of multiple data types
Limitations	Computationally intensive, steep learning curve	Amplicon only; large datasets may be slow	No raw sequence processing, depends on upstream pipelines
Best used for	Complete microbiome workflow from raw reads	Producing high-quality ASVs for downstream analysis	Statistical analysis, diversity assessment, plotting, and integrating data sources

QIIME2 (Quantitative Insights Into Microbial Ecology 2) is a next-generation, open-source, plugin-based microbiome bioinformatics platform that provides an end-to-end solution for analyzing marker-gene (e.g., 16S/18S/ITS) and shotgun metagenomic sequencing data. It is the successor to QIIME1, redesigned around the principles of modularity, reproducibility, and provenance tracking (Melounková et al., 2019).

DADA2 (Divisive Amplicon Denoising Algorithm 2) is a statistical pipeline designed to infer exact amplicon sequence variants (ASVs) from next-generation sequencing (NGS) data, such as 16S rRNA, 18S rRNA, or ITS amplicons, unlike traditional OTU clustering (which groups reads by arbitrary similarity thresholds, e.g., 97%), DADA2 models and corrects sequencing errors to distinguish true biological variants from artifacts with single-nucleotide resolution (Callahan et al., 2016).

**Figure 2:**
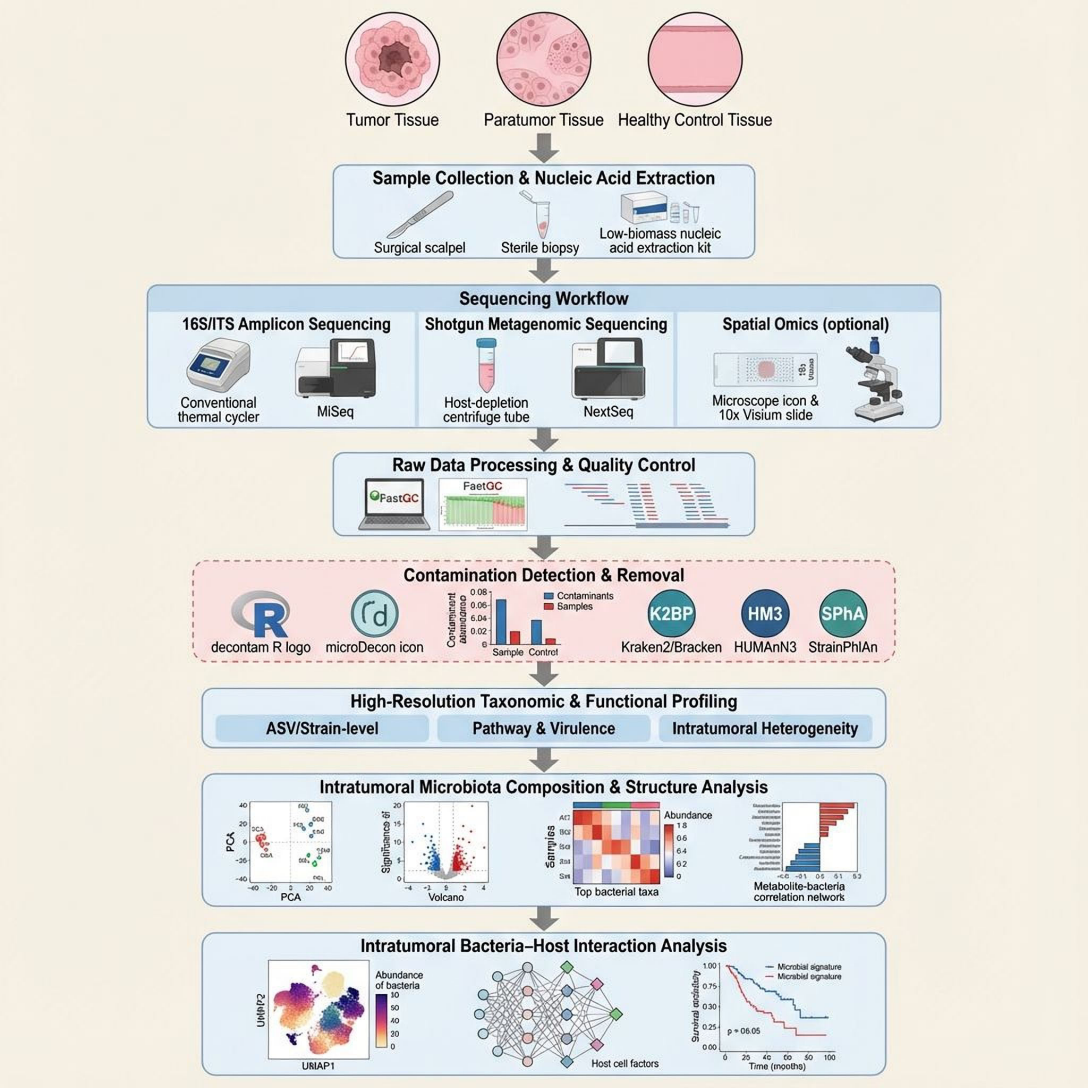
Overview of multi-omics sequencing workflows for characterizing intratumoral microbiomes. Tumor, paratumor, and healthy control tissues are collected and subjected to low-biomass–optimized nucleic acid extraction. Parallel sequencing strategies include 16S/ITS amplicon sequencing, shotgun metagenomics (with host depletion), and optional spatial omics. Raw data undergo quality control, host filtering, and rigorous contamination removal (decontam, microDecon, Kraken2/Bracken, HUMAnN3, StrainPhlAn). High-resolution profiling yields ASV/strain-level taxonomy, functional pathways, and intratumoral heterogeneity. The pipeline culminates in (i) intratumoral microbiota composition and structure analysis and (ii) bacteria–host interaction analysis.

Phyloseq is an open-source R package designed for the integration, analysis, and visualization of microbiome data. Rather than processing raw sequencing reads, Phyloseq operates on preprocessed data - typically ASV/OTU tables, taxonomic annotations, phylogenetic trees, and sample metadata - to enable comprehensive statistical and ecological interpretation of microbial community profiles (Sijtsema et al., 2014).

f. Use of controls for contamination monitoring: Every sequencing run must include a full panel of extraction blanks, air swabs, and mock communities. Their profiles serve as dynamic references against which any new contaminant signatures can be immediately recognized and subtracted (**Figure 2**).

## 4 Advanced techniques for enhancing detection and characterization of the intratumoral microbiota

The profound limitations of conventional bulk sequencing approaches in the context of ultra-low microbial biomass and marked spatial heterogeneity have necessitated the development and rapid adoption of substantially more sensitive and informative methodologies. The techniques outlined in this section, many of which have matured only within the past three to five years, now provide the resolution, specificity, and orthogonal validation required to progress from correlative taxonomic surveys to rigorous mechanistic and functional investigation of tumor-resident microbial communities (Chen et al., 2025).

### 4.1 Single-cell sequencing

Approaches such as INVADEseq selectively amplify bacterial 16S rRNA or universal prokaryotic transcripts within standard droplet-based human scRNA-seq libraries. This enables simultaneous profiling of the human transcriptome and bacterial presence/transcriptional activity in thousands of individual tumor and immune cells from the same specimen (Galeano Niño et al., 2022). Recent applications have definitively established intracellular residence of specific taxa (notably Fusobacterium nucleatum) in cancer cells and tumor-associated macrophages, while linking bacterial detection to distinct host transcriptional states associated with metastasis and immune suppression (Robinson et al., 2024). Flow-cytometric or microfluidic isolation of intact bacterial cells from enzymatically dissociated tumors, followed by low-input multiple displacement amplification or targeted 16S/whole-genome sequencing, dramatically reduces host-DNA background prior to library construction. Protocols such as CSI-Microbes achieve near-single-bacterium resolution and have confirmed widespread intracellular localization across multiple cancer types (Lloréns-Rico et al., 2022).

Despite their power, these techniques remain technically demanding and costly. They require high-quality fresh or flash-frozen tissue, specialized library preparation expertise, and substantial bioinformatics resources for dual human–microbial analysis. Detection sensitivity is also constrained by bacterial transcript levels; quiescent or low-rRNA organisms may be missed (Ke et al., 2022; Huang et al., 2023). Scaling to large clinical cohorts is currently impractical, and commercial clinical deployment remains distant (Nadukkandy et al., 2025).

### 4.2 Optimized functional experiments

The specific contributions of individual microbes in the TME can be studied via functional experiments. Optimized functional experiments are especially useful for investigating the interactions of low-biomass microbial communities with tumor and non-tumor cells. Effectively simulating the impact of physiologically relevant microbial loads in the tumor context poses one of the major challenges in this area. To ensure that microbial abundance remains low, yet stable, experimental designs should incorporate quantitative PCR and fluorescence in situ hybridization (FISH) to monitor dynamic microbial growth and metabolism (Emerson et al., 2017). Moreover, it is possible to adjust the composition and community structure of microbes by manipulating antibiotic pressure or culture conditions that simulate physiological low-biomass conditions. Studies should be carried out using high-throughput episodic detection of metabolites (short-chain fatty acids, bile acids), since the tumor’s metabolic profile can be modified by microbial metabolism (Nshanian et al., 2025). Furthermore, the assessment of immune response, characterization of tumor stem cells, and optimization of microbial and chemotherapeutic drug interactions will provide better insights into the function of low-biomass microbial communities (de Castilhos et al., 2024).

These techniques have moderate sensitivity to assess interactions or growth, but generally less than sequencing. In the microbiological context, all techniques indicate they are less sensitive than sequencing. FISH, for instance, may not be capable of capturing certain microbes without a known probe, limiting its power (Prudent and Raoult, 2019).

The experiment limitations include using physiologically relevant microbial loads in the tumor setting, and the infection is a challenge. Microbial populations are very complex *in vivo*. This complexity can easily be lost with experimental designs *in vitro*, leading to artificial findings (Du and Yang, 2024). Quantitative PCR and FISH are better suited to detect specific microbes. They are also extremely poor with respect to overall diversity (Feng et al., 2022).

Functional experiments are cost-effective relative to sequencing techniques, particularly for a narrow range of microbial species (M and Sebastian, 2021). However, the laborious nature of these experiments, specifically monitoring a microbe’s metabolism over a length of time, raises their overall costs more than would be apparent if they only interact with one species (Boggy and Woolf, 2010).

Although scalable in experimental settings, these methods do not have the high-throughput capabilities needed for clinical translation. Focused research, rather than routine clinical diagnostics, makes them the best fit for restraining clinical translation potential (Turjeman et al., 2025).

### 4.3 Culture-based methods

The combination of metabolic engineering and systems biology can help us to precisely optimize the metabolic pathways of microorganisms to improve biomass production (Choi et al., 2019).

High-throughput screening technology can accelerate the optimization of microbial growth conditions. Based on the nutritional requirements of microorganisms, it aids in the design of new media and the optimization of culture conditions, such as adjusting carbon, nitrogen, and trace element sources, as well as temperature and pH. Through parallel cultivation and analysis of a large number of samples, the optimal conditions affecting biomass can be discovered more quickly (Huber et al., 2011). In addition, automated culture platforms combined with data analytics allow real-time monitoring of growth status and timely adjustment of culture conditions. Recent studies have also shown that co-culturing between microorganisms (especially utilizing synergistic effects) can promote biomass growth. In a co-culture system, different microorganisms can complement their nutritional needs or synergize to break down complex organic matter, thereby increasing overall biomass production (Li et al., 2025).

Culture-based methods have less sensitivity to low-abundance or unculturable species that are present in the TME, as they can isolate or study specific strains of microbes (Steen et al., 2019).

One important limitation is that not all intratumoral bacteria are cultivable or grow under standard laboratory conditions. This prevents culture-based methods from accurately capturing the diversity of the intratumoral microbiota (Song et al., 2024).

Culture-based techniques do not require expensive equipment and materials, particularly for growing specific microbial strains. However, there are additional costs associated with high-throughput screening and automated culture platforms. Optimizing the conditions of culture can also be costly (Huang et al., 2023).

Culture-based approaches can be scaled up from small to medium studies, particularly for specific microbes. But they aren’t sensitive enough and can’t fully recreate natural microbiomes, making them of limited utility for obtaining a larger-scale representation of the entire intratumoral microbiota (Lobato et al., 2025). When attempting to isolate known pathogens or microbes that associate with a specific tumor type, culture methods may have clinical translation. Nonetheless, their application is constrained because they fail to capture all microbial species, especially the non-cultured ones, restricting their use in clinical microbiome diagnostics (Lewis, 2013).

### 4.4 Multiple-modality integrated framework

Employing several analytical techniques can yield a holistic understanding of the intratumoral microbiota. The analysis of genomic and transcriptomic information is essential equipment for microbial community studies (Baldrian and López-Mondéjar, 2014). The 16S rRNA gene sequencing analysis of the intratumoral microbiota and RNA-seq analysis can examine the microbiota’s gene expression in tumors, its role in the TME and tumor progression, and its impact on therapeutic response (Zhou et al., 2024). It is also possible to combine 16S sequencing with macrogenomics to simultaneously obtain species- and functional-level information about the microbiota and explore how their metabolic activities affect tumor growth or immune escape. Metabolomic profiling of colony metabolites (short-chain fatty acids), combined with immunobiological data (immune cell infiltration), can be used to assess the colon’s role in immune microenvironment modulation. The use of integrated approaches enhanced detection sensitivity by providing cross-validation among different techniques to not miss low-abundance species.

### 4.5 Key analysis of spatial localization of intratumoral bacteria

Spatial transcriptomics has emerged as an indispensable tool for delineating the topographic organization of microbial communities within the TME and linking their distribution to local host transcriptional programs.

A seminal study employing 10x Visium spatial transcriptomics as its core platform demonstrated that intratumoral bacteria are not stochastically dispersed but exhibit distinct spatial patterning that correlates strongly with regional gene-expression signatures and cellular phenotypes (Desai et al., 2024). Unlike bulk transcriptomic approaches, which forfeit all positional information, this technology preserves spatial context at near-cellular resolution, thereby revealing previously obscured host–microbe interactions.

Subsequent landmark investigations have combined high-resolution spatial transcriptomics with complementary modalities (in situ hybridization using 16S rRNA-targeted probes and single-nucleus or single-cell RNA sequencing) to map bacterial localization with unprecedented precision (Galeano Niño et al., 2022). In oral squamous cell carcinoma and colorectal adenocarcinoma, these integrated analyses showed that bacteria preferentially colonize discrete tumor subregions and specific cell types. Regions of bacterial enrichment were consistently characterized by pronounced immunosuppressive transcriptional states, including expansion of CD66b^+^ granulocytic populations and elevated expression of immune-checkpoint molecules (CTLA4, PDCD1). Bacterial presence was further associated with reduced tumor-cell proliferation, disrupted epithelial–immune crosstalk, and molecular signatures favoring immune evasion and metastatic propensity (Deng et al., 2023).

The sensitivity of current spatial transcriptomics platforms for detecting intratumoral bacteria now rivals or exceeds that of optimized bulk metagenomics, while simultaneously providing critical anatomical context that bulk methods cannot resolve (Takano et al., 2024).

Nevertheless, important limitations persist. Contemporary high-resolution platforms require specialized instrumentation, substantial computational infrastructure, and considerable per-sample expenditure, rendering them labor-intensive and prohibitively costly for large clinical cohorts (Deng et al., 2023). These constraints currently confine their application to hypothesis-driven mechanistic studies of selected tumor types or regions rather than routine diagnostic or prognostic use (Marx, 2021). Despite these hurdles, the translational potential of spatial multi-omics remains substantial. By elucidating spatially restricted microbe–host interactions that govern local immune tone, therapeutic response, and metastatic niches, these approaches are poised to inform rationally designed, personalized interventions once technical and economic barriers are surmounted. At present, however, clinical implementation remains limited to research settings.

### 4.6 Concluding comparison and integration of advanced methodologies

Contemporary spatial and single-cell multi-omics platforms substantially outperform conventional culture-based methods, 16S rRNA amplicon sequencing, and bulk metagenomics in intratumoral microbiota research. By preserving tissue architecture, these approaches eliminate averaging artefacts and map microbial identity, viability, and activity at near-cellular resolution alongside host transcriptional, proteomic, and metabolic profiles.

This resolution enables precise discrimination of intracellular, stromal, and extracellular localization, confident exclusion of contaminants, and detection of niche-specific colonization patterns. Integration with *in situ* transcriptomics, proteomics, and metabolomics elucidates active microbial pathways, induced host signaling, and local metabolite gradients that regulate immune-cell function in discrete tumor subregions, markedly reducing false positives inherent to low-biomass studies.

These datasets facilitate robust mechanistic hypotheses regarding microbial roles in immunomodulation, metabolic reprogramming, and therapy resistance, while revealing spatially restricted biomarkers and neighborhood-specific therapeutic targets.

In conclusion, single-cell sequencing, advanced anaerobic cultivation, spatial multi-omics, and integrated multi-modality frameworks collectively overcome low-biomass and contamination challenges. Despite differing in sensitivity, cost, and scalability, their combined, standardized application now represents the most reliable strategy for defining the diversity, localization, and functional impact of intratumoral microbiota, paving the way for translation into clinical oncology.

## 5 Applications of anaerobic microbial research in intratumoral microbiota studies

### 5.1 New technologies for anaerobic culture

Recent breakthroughs in anaerobic cultivation systems are fundamentally transforming our capacity to isolate and functionally interrogate the strictly anaerobic and microaerophilic bacteria that predominate in many solid tumors. For decades, the extreme oxygen sensitivity and complex nutritional requirements of these organisms rendered the vast majority unculturable using conventional agar-plate or broth methods, severely limiting mechanistic studies. The new generation of technologies outlined below now permits routine recovery of previously intractable taxa from hypoxic tumor cores, thereby bridging the critical gap between sequencing-based discovery and experimental validation.

a. Advanced anaerobic chambers and robotic automation: Modern anaerobic chambers equipped with palladium catalysts, real-time O_2_/H_2_/CO_2_ sensors, and robotic arms maintain strict anoxia (<0.1 ppm O_2_) while enabling high-throughput plating, colony picking, and phenotypic screening with minimal human intervention (Graves et al., 1988; Askari et al., 2025). These systems dramatically reduce inadvertent oxygen exposure and have substantially increased isolation success rates for obligate anaerobes from pancreatic, colorectal, and breast tumors.

b. In situ cultivation devices (“iChip” technology): Devices that permit microbial growth within semi-permeable membranes while embedded in native tumor tissue or implanted in gnotobiotic hosts exploit in situ nutrient and signal exchange, allowing recovery of organisms dependent on tumor-specific metabolites and redox conditions that cannot be replicated in vitro (Chabib et al., 2025).

c. Genome-guided and AI-optimized synthetic media: Metagenome-assembled genomes from tumor specimens are used to predict autotrophies and essential cofactors (haem, menaquinone, short-chain fatty acids, amino-acid derivatives). Custom chemically defined or semi-defined media, iteratively refined by machine-learning algorithms, now routinely support primary isolation of fastidious anaerobes that fail on rich complex media (Laso-Pérez et al., 2018; Burgos da Silva et al., 2022).

d. Anaerobic microfluidic and droplet-based systems: Picolitre-scale droplets or nano wells maintained under strict anoxia, combined with computer-vision-based growth detection and automated sorting, enable parallel screening of thousands of micro-environments and single-cell isolation of rare tumor-associated strains (Burgos da Silva et al., 2022; Niu et al., 2024).

These advances have already yielded viable isolates of Fusobacterium, Clostridium, Bifidobacterium, Peptoniphilus, and other genera repeatedly implicated in tumor progression and immunomodulation. The availability of pure cultures now facilitates controlled infection of patient-derived organoids, gnotobiotic mouse colonization, and precise dissection of metabolic and immune crosstalk-capabilities that were previously unattainable. Continued refinement of these cultivation platforms is therefore essential for transitioning intratumoral microbiome research from correlative taxonomy to rigorous causal and translational investigation.

### 5.2 Soft method enhancements and AI integration

In summary, the efficiency and simplicity of cultivating anaerobic microbes will greatly improve with AI. This technology will take the lead in better screening and harnessing many functions of technology. Research is a field of rapid development. As we move away from sequencing-based profiling and into functional microbiomics, we will need increased ability to culture and manipulate the many anaerobic microbes to reveal their role in cancer development and treatment. The tissue of some tumors contains hypoxic (low oxygen) regions that may serve as a niche for anaerobic and facultative anaerobic colonizing microbes. Microbes can survive and grow in poorly vascularized tumor cores that are hypoxic, such as those found in pancreatic cancer, colorectal cancer, and breast cancer (Zhou et al., 2024). In anaerobic microbiology, AI moves to experiment orchestration from data processing (Kumar et al., 2025). According to the study, closed-loop systems that integrate the cultivation of marine microbes, phenotyping, and model updating will allow AI to progressively refine anaerobic growth conditions (Capela et al., 2025). By the application of reinforcement learning, optimized media compositions for previously uncultivated strains have been designed, while coupling with microfluidics cultivation and computer vision generates high-density phenotypic data for the training of predictive models for growth dynamics and stress responses (Gong et al., 2022). AI with automated culturomics platforms enables even greater throughput; robotic inoculation, environment control, and high-resolution imaging allow the establishment and screening of thousands of parallel anaerobic cultures with minimal oxygen exposure (Coutant et al., 2019).

## 6 AI-driven analysis of intratumoral strain function and metabolic characteristics

### 6.1 AI applications in whole-genome analysis for microbiome research

The utilization of AI in microbiomics, specifically for functional prediction and whole-genome analysis of bacterial gene elements, has greatly enhanced our comprehension of microbes’ functions in the TME. Using sequence alignment and annotations available in databases, traditional genomics has been unsuccessful in dealing with unannotated genes and Structural Variations. On the other hand, AI algorithms, especially deep learning and graph neural networks (GNNs), allow the automatic learning of latent representations in genomic data to classify gene elements and functional regions better (Hasibi et al., 2024). Examining the arrangement of genetic material enables the identification of new elements, which contribute in important ways to the TME.

Working with multi-omics data (genomic, transcriptomic, etc.) to analyze the tumor microbiome in unison is also beneficial to AI. By building complex networks of gene-gene and gene-environment interactions, AI can help understand the function of bacterial genes in the biological context of tumors. The use of AI technologies, such as GNNs, in functional analysis of bacterial gene elements. With the help of these techniques, bacterial genes can be annotated for their functions, and their possible roles in the tumor microbiome can be predicted. This particularly pertains to the link of genes with tumor/immune cells of bacterial origin (Zhang et al., 2021). Moreover, AI is instrumental in detecting drug resistance genes and their functional elements in the tumor microbiome, which offers key insights into tumor resistance mechanisms (Mao et al., 2025).

### 6.2 AI-enabled investigation of microbial metabolic enzymes

AI is playing a bigger role in microbial functional genomics, especially in finding metabolic enzymes and discovering tumor-microbiome interactions (Kumar et al., 2025). Protein language models now permit the inference of enzymatic function directly from the sequence space, further enabling the detection of divergent enzymes or uncharacterized metabolic pathways beyond the traditional homology-based annotation of enzymes (Capela et al., 2025). Deep learning frameworks, such as graph-based models, can analyze integrated microbe-metabolite-host networks and pinpoint metabolic nodes associated with tumor progression (Gong et al., 2022).

Adoption of advanced Genomics, Metabolomics, and Structural Biology, along with computational methods for predicting enzyme functions, is being studied with the insights of microbial metabolic networks. A skeletal bile acid, 3-acetoDCA, that may represent a new therapeutic target in metabolic diseases was discovered through computational analysis (Ding et al., 2025). The researchers discovered that microbial enzymes were involved in tumoral metabolic processes and that their metabolic products were able to regulate host immune responses. Since microbial metabolic enzymes are immune modulators, it is possible to study their relationship to better understand how they induce tumoral immune evasion. For instance, by regulating immune checkpoint activities and/or modifying immune cell infiltration (Queen et al., 2023).

Furthermore, there have been considerable advances in the development of targeted therapies for microbial metabolic enzymes. Research suggested that microbial metabolites can enhance immunotherapy efficacy through T-cell stemness reprogramming in pan-cancer patients (Jia et al., 2024). Also, the use of computational tools and techniques helps to find potential inhibitors, optimize drug formulations, and improve bioavailability and therapeutic outcome (Wang et al., 2024).

## 7 Organoids as a frontier model in intratumor microbiome research: applications and advantages

A major obstacle in tumor microbiome research is the lack of suitable in vitro models to investigate the pathogenic role and mechanisms of intratumoral microbiota. This is primarily due to two key reasons:

Bacterial Overgrowth Under Standard Culture Conditions: Even at a low multiplicity of infection, facultative anaerobic bacteria proliferate rapidly under conventional cell culture conditions (aerobic environment), leading to overwhelming bacterial expansion and subsequent tumor cell death within a short period (Roszkowska et al., 2024). This makes it impossible to replicate the true in vivo intratumoral bacterial microenvironment.

Indirect Mechanisms Mediated by the TME: Most intratumoral bacteria exert their effects on tumors not through direct interaction with cancer cells, but rather by modulating components of the TME, such as immune cells and stromal cells (Yu et al., 2024). Thus, simplistic tumor cell-bacteria co-culture models fail to capture the multidimensional interactions among bacteria, the TME, and tumor cells.

The application of organoid models could effectively overcome the aforementioned challenges in tumor microbiome research (Chen et al., 2022).

Organoids have become an important tool in cancer research, especially in TME and tumor microbiome studies. There are many existing examples of organoid models applied in the study of infectious diseases. For example, researchers have constructed differentiated human intestinal organoids by extracting stem cells from healthy human small intestinal tissues and adding facilitators, such as bile acids, to the culture medium to enhance viral entry and replication. The researchers successfully replicated human norovirus strains GII.4 and GII.3 in the organoids, which solved the technical problem that norovirus could not be cultured in vitro for a long time (Hayashi et al., 2024). Another study extracted human gastric mucosa tissue or iPSCs to obtain gastric organoids and exposed the organoids to H. pylori. The results showed that the infection induced the organoids to secrete a large amount of IL-8 and IL-1β, which suggested the activation of the inflammatory response, and the long-term exposure induced intestinal metaplasia and gastric precancerous lesions (Westmeier et al., 2018).

Organoids, an ex vivo three-dimensional (3D) culture model, more accurately recapitulate the structure, heterogeneity, and function of clinical tumors compared to conventional tumor cell cultures. In cancer research, organoid technology has emerged as a transformative tool for investigating the interactions between tumor cells and stromal cells (Dawoody Nejad and Pioro, 2025). Increasingly, research attention has focused on the tumor microbiome, which is increasingly recognized to play a role in tumorigenesis, progression, immune evasion, and therapeutic response. Organoid technology is a promising and physiologically relevant model to study the host–microbe interactions. An area of interest is the relationship between intertumoral bacteria and the TME. **Table 4** summarizes some organoid models used to study intratumoral pathogen-host interactions.

**Table 4:** Applications of organoid models in studying intratumoral pathogen–host interactions.

Pathogen	Organoid Model	Traditional *In Vitro* Model	Key Findings	Key Advantages of Organoids	Citation
*Clostridium*	Human intestinal enteroids	Two-dimensional cell culture	Clostridium difficile accumulates in the TME and mediates resistance to 5-fluorouracil by inducing cell cycle arrest.	*In vitro* construction of microstructures that highly mimic *in vivo* tissues	(Galeano Niño et al., 2025)
*Staphylococcus aureus*	Human skin organoids	HaCaT keratinocyte monolayers	Demonstrated biofilm formation, epithelial disruption, and cytokine induction in stratified skin-like layers.	Mimics epidermal stratification and natural wound architecture.	(Liu et al., 2025)
*Helicobacter pylori*	Human gastric organoids	AGS gastric cancer cells	Demonstrated *H. pylori* activates NF-κB and IL-8 in a spatially dependent manner in human-like gastric epithelium.	Recapitulates gastric gland structure, epithelial polarity, and native receptor expression.	(Uotani et al., 2019)
*Helicobacter pylori*	Human gastric organoids	Primary gastric cells (short-lived)	*H. pylori* targets Lgr5+ gastric stem cells and promotes their expansion via Wnt signaling activation.	Maintains stem cell niche, enables study of stem–pathogen interactions.	(He et al., 2023)
*Salmonella enterica*	Mouse intestinal organoids	Caco-2, HT-29	Salmonella effector AvrA inhibits autophagy and enhances epithelial invasion; organoids showed suppressed LC3 expression.	Captures host defense signaling (autophagy), epithelial polarity, and inflammation better than 2D models.	(Copland et al., 2024)
*Salmonella Dublin*	Bovine ileum-derived intestinal organoids	Bovine primary epithelial cells	Demonstrated epithelial barrier disruption and IL-8 secretion in response to zoonotic *Salmonella*.	Veterinary infection model, relevant for translational zoonotic disease research.	(Kawasaki et al., 2024)
*Mycobacterium tuberculosis*	Human alveolar type II (AT2) organoids	A549, THP-1 macrophage co-culture	Revealed early epithelial damage, necrosis, and granuloma-like structures; Mtb invaded and replicated in AT2 cells.	Models lung epithelial remodeling and host–pathogen interactions; includes non-immune and immune components.	(Armet et al., 2022)
*Norovirus*	Human intestinal enteroids	None (could not replicate virus)	First *in vitro* replication of human norovirus; replication was genotype- and bile acid-dependent.	Fully functional enterocytes and goblet cells; supports infection that 2D systems failed to replicate.	(Ettayebi et al., 2016)
*Zika virus*	Human brain organoids	Neural stem cell monolayers	Revealed Zika-induced microcephaly phenotype: reduced organoid size, increased cell death, impaired neurogenesis.	Captures fetal brain development stages; essential for neurotropism and microcephaly modeling.	(Garcez et al., 2016)
*SARS-CoV-2*	Human bronchial and alveolar organoids	Vero E6, Calu-3	Showed tissue-specific viral tropism; alveolar cells were more permissive; it demonstrated cytokine production and viral replication kinetics.	Maintains lung-specific cell types (AT2, ciliated, basal cells); used for drug screening and immune response analysis.	(Peckham et al., 2020)

### 7.1 Applications of organoids in tumor microbiome research

a. Modeling microbe–tumor interactions in 3D: By co-culturing tumor organoids obtained from patients with defined intratumoral microbes, including anaerobic bacteria, real-time monitoring of colonization and invasion of tumor-mimicking structures is achieved. Moreover, this model enables the analysis of the interactions of strains with tumor and stromal cells (Dutta et al., 2017). This approach helps in gaining insight into host-microbe interactions inside the TME.

b. Immune-enriched organoids for host–microbiota crosstalk: Immune organoids that include tumor-infiltrating lymphocytes or autologous immune cells can be used to study how intratumoral microorganisms affect the immune TME (Moraitis et al., 2025; Wagoner et al., 2025). This is crucial for comprehending the immune modulation caused by gut bacteria as well as the response to immunotherapies such as immune checkpoint blockade (Martín-Arana et al., 2025; Röder et al., 2025).

c. Drug response and resistance modeling: There is evidence that co-culturing tumor organoids with defined microbiota can reveal if bacterial metabolites or microbial enzymes impact response to chemotherapy or immunotherapy (Wang et al., 2018). Important discoveries of microbiome-driven drug resistance mechanisms have been made, along with the potential for microbiota-informed therapeutics (Peng et al., 2025; Zhang et al., 2025).

d. Personalized microbiome–tumor profiling: Patient-derived organoids (PDOs) can be used to test the functional impacts of an individual’s intratumoral microbes. This opens personalized investigations of microbiome-tumor interactions and the use of microbiome modulation as part of personalized medicine (Sayed et al., 2024; Tao et al., 2025).

### 7.2 Advantages of organoids over traditional models

Human Relevance: Tumor organoids maintain patients’ genome and phenotype divergence from their murine counterparts, exaggerating their actual disease in patients (Howell et al., 2018; Andreatta et al., 2025).

a. Long-term co-culture stability: Advanced organoid culture technologies can be effectively used for prolonged co-culture with facultative as well as obligate anaerobes under well-controlled simulated hypoxic conditions (Ng et al., 2019; Kuse et al., 2025). This is a well-established in vitro model that supports tumor-like oxygen gradients, which result in microbiota colonization and metabolite exchange, which preserves epithelial integrity. Furthermore, these systems also allow us to study the transcriptional and metabolic remodeling induced by microbes over longer periods of observation.

b. Scalability and manipulability: Organoids have the potential to be developed on a large scale and altered genetically. More importantly, they may be taken through single-cell or spatial omics to study host interactions (Ishahak et al., 2025; Zhao et al., 2025). This flexibility enables a detailed investigation into the impact of specific microbes on tumor immunity, metabolism, and differentiation. Microfluidic integration facilitates the regulation of additional environmental and microbial parameters in a controlled manner.

c. Ethical and practical superiority: Organoids reduce the need for experiments on animals while also enabling experimentation on patient-specific organoids, which would be impossible to replicate in vivo (Schäffers et al., 2025; Verstegen et al., 2025). According to the 3R Principles (Replacement-Reduction-Refinement), organoid-based systems are compliant as they reduce our reliance on animal testing while also being a more human-relevant and reproducible platform.

By integrating organoid technology with microfluidic organ-on-chip systems, synthetic microbiota consortia, and multi-omics profiling, we will be further advancing the insights into the tumor-microbiome ecosystems. In the end, co-culture models of organoid microbes have the potential to identify key therapeutic targets (Taş et al., 2025; Zhan et al., 2025).

### 7.3 Limitations of organoid models

Researchers continue to make progress in synthetic media as well as chemically defined culture conditions. Nevertheless, present organoids lack immune, vascular, and neural components, which could undermine cell identity and functionality. Co-culture and pluripotent stem cell-derived models increase cell diversity but are developmentally immature (Cordero-Espinoza et al., 2021); while in vivo transplantation enhances organoid maturation but decreases experimental controllability (Takebe and Wells, 2019).

Organoids poorly reproduce physiological oxygen gradients. They have core hypoxia, which is due not to dynamic regulation but to diffusion limits. This hampers the modeling of metabolism and host-microbiome interactions (Wang et al., 2024). The self-organizing properties also result in high variability in morphology and differentiation, which hinders reproducibility and scalability.

### 7.4 Perspectives and future directions for organoid-based tumor-microbiome research

Despite their considerable promise, patient-derived organoids, microfluidic tumor-on-chip platforms, and defined synthetic microbial consortia remain subject to important limitations that must be critically addressed before widespread mechanistic or translational application can be realized. Current organoid systems typically lack full immune-cell diversity, vascular networks, and innervation, resulting in incomplete recapitulation of the native TME. Maintaining physiologically relevant microbial consortia over extended periods without overgrowth or loss of key taxa poses additional challenges, while high per-experiment costs and limited throughput constrain scalability.

Nevertheless, these models currently offer the closest approximation of in vivo spatial architecture, oxygen gradients, and cell–cell interactions achievable ex vivo. When judiciously combined with spatial multi-omics readouts, anaerobic cultivation-derived isolates, and rationally designed microbial consortia, they provide a powerful framework for dissecting causal microbe–tumor–immune relationships at previously unattainable resolution.

Ultimately, progress in intratumoral microbiome research will depend not only on continued technological refinement but, more critically, on conceptual and experimental redesign that prioritizes physiological fidelity, rigorous standardization, and multi-modal integration. Only through such holistic, interdisciplinary strategy—merging advanced culture systems, high-resolution analytics, and reproducible model platforms—can the field generate robust, scalable, and clinically translatable insights capable of bridging the persistent gap between observational discovery and therapeutic implementation.

## 8 Leveraging the tumor microbiome for clinical research and therapeutics

An important area for future clinical cancer research is intratumoral microbial studies, especially those involving clostridia. Anaerobic bacterial growth in tumors could help researchers better understand the mechanisms of tumor development and progression, including immune regulation and drug resistance (Niu et al., 2024). The ability to isolate and harvest such strains, or the availability of their cultured counterparts, can be used to conduct functional studies that confirm their biological role, along with intratumoral bacterium or their metabolites as possible biomarkers or therapeutic targets in precision oncology (Burgos da Silva et al., 2022).

When AI combines with microbiome and clinical multi-omics data, it could drive a new era in cancer research and application. Machine learning can reveal how intratumoral microbiomes impact metastasis and treatment response. It can identify microbial biomarkers and track microbiome-mediated tumor evolution (Cheung and Yu, 2021). Illiani et al. used 16S rRNA sequencing with Penalized Logistic Regression Analysis (PELORA) to find bacterial clusters distinct between metastatic and non-metastatic samples and then applied Iterative Random Forest to correlate microbial abundances with metastasis risk (Villani et al., 2024). Similarly, Ma and others also made use of AI to assess the intestinal microbiome of CRC patients for over 40 anti-cancer peptides (ACPs) that were newly discovered (Ma et al., 2023).

AI is also used in the early detection and treatment of cancer. AI-based predictive models have the potential to determine whether healthy individuals are at risk of developing certain cancers by examining microbiome signatures in blood, urine, or stool samples (Han and Lee, 2024). Research has shown that AI-assisted analysis of a patient’s urine microbiome can help detect an early stage of bladder cancer (Lee et al., 2025). Apart from this, AI can also help come up with microbiome-guided therapies. For instance, in one case, AI helped the cancer immunotherapy treatment by predicting the best microbial combinations for enhancing the synergistic effect of an immune checkpoint inhibitor. This was done to treat lung cancer in specific models (Routy et al., 2018).

The use of AI in intratumoral microbiome research is enabling new insights into cancer progression, as well as the discovery of new biomarkers and therapeutic targets. With the advancing technology, these great methods can completely change cancer diagnosis, prognosis, and personalized treatment.

## 9 Future perspectives

### 9.1 From description to mechanism and targeted intervention

Intratumoral microbiome research is shifting from descriptive profiling and correlative associations toward rigorous mechanistic dissection and precision therapeutics (Wong-Rolle et al., 2021; Boesch et al., 2022; Bahuguna and Dubey, 2023; Chen and Huang, 2024; Wang et al., 2025). Future progress demands experimental systems that establish causality, define molecular pathways of microbial influence on tumor biology and immunity, and enable rational, microbiota-directed interventions.

### 9.2 Technological advancement as a foundation

Intratumoral microbiome studies face extremely low-biomass, near-absent signal in some tumors, high contamination risk, and the need for single-cell/subcellular resolution. Advances will depend heavily on ultra-sensitive long-read sequencing, multiplexed 16S/meta transcriptomics (Cheng and Wang, 2025), high-resolution in situ imaging of bacteria (Urban et al., 2021), and integrated multi-omics platforms that simultaneously capture microbial and host responses (Chen et al., 2021; Abe et al., 2024).

### 9.3 Deep interdisciplinary integration

The intricate interplay among microbes, tumor cells, and immune effectors demands sustained collaboration across microbiology, oncology, immunology, and systems biology to elucidate microbe-driven mechanisms of oncogenesis, immune modulation, and therapeutic resistance (Bohm et al., 2025). Progress will further require integration of artificial intelligence, network modelling, and dynamic simulations to reconstruct multilayered microbe-host-tumor interaction networks (Zhang et al., 2025), alongside systems-level analyses of microbiome-mediated immune escape, tumor heterogeneity, and loco-regional or systemic inflammation (Ling et al., 2019; Zhang et al., 2024).

### 9.4 Standardization, open science, and reproducibility

Credible cross-study comparisons require urgent standardization of sample collection, DNA extraction, sequencing, and bioinformatics protocols (Shen et al., 2022). Establishing public repositories with comprehensive metadata (tumor type, host genetics, treatment history) (Ahlinder et al., 2022) and fostering open sharing of raw data, pipelines, and protocols are essential to ensure reproducibility, transparency, and accelerated progress in the field.

Advances in detection technologies, close cooperation between disciplines, as well as standardized, interoperable, and reproducible data ecosystems, will shape the future of intratumoral microbiome research. To translate microbial findings into applications, we need an open and trustworthy scientific infrastructure that will help advance the field of microbial cancer research and redefine tumor biology.
